# Luteolin and its derivatives: modulation of epithelial-mesenchymal transition in fibrosis and cancer

**DOI:** 10.3389/fphar.2026.1841533

**Published:** 2026-07-06

**Authors:** Guodong Zang, Ying Liu, Xingyu Mu, Yingju Li, Rui Fan

**Affiliations:** 1 Department of Respiratory and Critical Care Medicine, Shandong University of Traditional Chinese Medicine Affiliated Hospital, Jinan, Shandong, China; 2 Department of Pulmonary Medicine, Xiangyang Hospital affiliated to Hubei University of Chinese Medicine, Xiangyang, Hubei, China; 3 Department of Clinical Laboratory, Shandong Provincial Hospital affiliated to Shandong First Medical University, Jinan, Shandong, China; 4 Respiratory and Critical Care Medicine Department, Shandong Provincial Hospital Affiliated to Shandong First Medical University, Jinan, Shandong, China

**Keywords:** cancer, ECM, EMT, fibrosis, flavonoids, luteolin

## Abstract

**Background:**

Fibrosis and cancer share epithelial-mesenchymal transition (EMT) as a core pathological driver, making EMT-targeted therapy a promising dual strategy. The natural flavonoid luteolin shows preliminary anti-fibrotic and anti-tumour activities, but its translational potential remains poorly systematized.

**Methods:**

We conducted a ConPhyMP compliant systematic review, searching PubMed for 2008–2026 studies on luteolin and its derivatives, EMT, fibrosis and cancer, to synthesize evidence on their mechanisms, efficacy and translational prospects.

**Results:**

Luteolin inhibits EMT via multi-target regulation of core pathways (TGF-β1/Smad, PI3K/Akt, Hippo/YAP), exerting consistent dose-dependent efficacy in suppressing cancer metastasis and multi-organ fibrosis. Structural modifications and nanodelivery systems resolved its bioavailability limitations, and rigorous validation ruled out pan-assay interference compounds (PAINS).

**Conclusion:**

Luteolin and its derivatives are promising dual-effect EMT-targeted therapeutics. Optimized formulations lay the groundwork for clinical translation, and future large-scale trials are needed to validate their clinical efficacy.

## 1 Introduction

Fibrosis and cancer represent significant global health challenges, sharing complex and intertwined pathological mechanisms despite their distinct clinical manifestations (Chen B. et al., 2025; Baniasadi et al., 2024; Luo et al., 2026). Cancer is characterized by uncontrolled cellular proliferation, invasion, and metastasis, leading to substantial morbidity and mortality globally (Mahdessian et al., 2021). For instance, the morbidity and mortality rates of tracheal, bronchial, and lung cancers remain persistently high worldwide, with distinct epidemiological patterns shaped by age and sex (Zhou et al., 2022; Xu Q. et al., 2025). Globally, tumors including breast cancer, lung cancer, prostate cancer, and gastrointestinal cancers contribute significantly to the burden of disease (Mubarik et al., 2024; Lin et al., 2019). Soft tissue sarcomas also represent a notable global health concern (Zhou J. et al., 2025). Fibrosis, on the other hand, is defined by the excessive accumulation of extracellular matrix (ECM) proteins, leading to tissue stiffening, organ dysfunction, and eventual organ failure (Dees et al., 2021; Di et al., 2025; Chu and Quan, 2024). This pathological process underlies a wide range of chronic fibrotic diseases such as the liver fibrosis, renal fibrosis, cardiac fibrosis, exemplified by myocarditis, and pulmonary fibrosis (Dees et al., 2021; Nauf et al., 2024; Li L. et al., 2025). For example, pulmonary fibrosis, particularly idiopathic pulmonary fibrosis (IPF), is a lethal disease with a median survival of less than 4 years, often escalating the risk of lung cancer (Chen B. et al., 2025).

Although fibrosis and cancers are traditionally regarded as distinct pathological entities, accumulating evidence reveals substantial mechanistic overlap between them (Chen B. et al., 2025; Baniasadi et al., 2024; Luo et al., 2026). Both conditions are driven by dysregulation of cell signaling pathways that lead to an altered cellular microenvironment. The transforming growth factor-beta 1 (TGF-β1) signaling pathway is a key common mechanism that is essential to both fibrogenesis and carcinogenesis (Luo et al., 2026; Chen F. et al., 2025; Deng et al., 2024). TGF-β1, upon interacting to its receptor, activates both canonical (Smad2/3-dependent) and non-canonical pathways, facilitating profibrotic processes including fibroblast activation, EMT, excessive ECM formation (e.g., collagen), and inhibition of matrix breakdown (Chen F. et al., 2025). EMT is a critical process in both fibrosis progression and cancer metastasis, allowing epithelial cells to acquire mesenchymal characteristics, enhancing their migratory and invasive capabilities (Chen F. et al., 2025).

Fibroblast activation, resulting in differentiation into myofibroblasts, is a characteristic feature of both fibrotic disorders and the tumor microenvironment (TME) (Younesi et al., 2024). Myofibroblasts are defined by the *de novo* expression of alpha-smooth muscle actin (α-SMA) and serve as central mediators of ECM deposition and tissue contraction. In cancer, these activated fibroblasts are termed cancer-associated fibroblasts (CAFs), which actively remodel the ECM into a stiff, aligned scaffold that promotes tumor cell invasion and metastasis (Cox, 2021). This modified ECM serves both as a physical barrier and as a signaling center, affecting cellular behavior via mechanotransduction. The stiffened matrix, a consequence of increased collagen cross-linking, provides aberrant mechanical cues that promote cancer cell proliferation and survival, often via pathways like the Hippo pathway effectors YAP and TAZ (Chu and Quan, 2024; Wei et al., 2023; Xiaojun et al., 2025; Dey et al., 2020).

**FIGURE 1:**
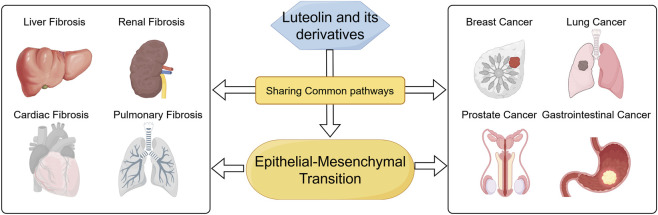
Luteolin and its derivatives participate in fibrosis and cancer progression through the EMT mechanism. By Figdraw.

Based on these findings, we posit that elucidating the intricate interactions between epithelial and mesenchymal cells is essential. Given the central role of EMT in the progression of fibrosis and cancer, targeting this process gradually become a promising therapeutic strategy. Flavonoids, including baicalin, casticin, quercetagitrin, GL-V9, and luteolin are a category of polyphenols prevalent in plants that demonstrate both anti-fibrotic and anti-cancer properties via a shared mechanism that regulates inflammation, oxidative stress, cell proliferation, apoptosis, and extracellular matrix remodeling (Wenbo et al., 2024; Mazurakova et al., 2024). Luteolin, a natural flavonoid, is increasingly demonstrating potential in counteracting both diseases through the modulation of EMT (Imran et al., 2019). This study commences with a categorization of flavonoids and clarifies the molecular mechanisms via which luteolin and its derivatives modulate EMT. We specifically highlight their dual capacity to impede fibrosis and cancer progression (Figure 1).

## 2 Materials and methods

This systematic review was conducted in full compliance with the ConPhyMP guidelines for phytopharmacological research (Heinrich and Jalil, 2023; Jalil et al., 2026). All included plant materials were classified as Extract Type A. Chemical characterization of extracts followed official pharmacopoeial standards, using validated HPLC-DAD methods with at least three detection parameters and chromatographic fingerprinting; at least two marker compounds were quantified with clear justification, and all analyses employed official reference standards for direct chromatographic overlay and comparison. All primary studies adhered to the Nagoya Protocol, CITES and relevant phytosanitary regulations, and commercial reagents and extracts were accompanied by certificates of analysis detailing batch numbers, purity and manufacturer information.

We conducted a literature search on PubMed using the following search strategies (luteolin OR luteolin derivatives) AND (epithelial-mesenchymal transition OR epithelial-mesenchymal transition OR EMT) AND (fibrosis OR cancer). The included studies spanned from 2008 to 2026.

## 3 Classification of flavonoids

Flavonoids constitute a vast and diverse class of plant-derived polyphenolic compounds, unified by their characteristic C6-C3-C6 carbon skeleton, comprising two benzene rings (A and B) linked by a three-carbon heterocyclic ring (C-ring) (Krishna et al., 2025). The systematic classification of flavonoids is primarily dictated by the degree of oxidation and saturation of the C-ring, the presence or absence of a C2-C3 double bond, the substitution pattern of hydroxyl groups, and the position of the B-ring attachment (Xu Y. et al., 2025). Flavonoids are systematically categorized into seven primary subclasses based on structural changes in their core carbon skeleton: flavonols, flavones, isoflavones, anthocyanins, flavanones (Mumtaz et al., 2026), flavanols, chalcones, and Dihydroflavonols. The classification and structural characteristics of flavonoids are shown in Table 1.

**TABLE 1:** Classification and structural characteristics of flavonoids.

Subclass	C-ring structural features	B-ring position	Biological highlights	Representative compounds
Flavones	C2-C3 double bond; no C3-OH	C2	Mitigates fibrosis via TGF-β/Smad, STAT3, NF-κB, AMPK	Luteolin, Apigenin
Flavonols	C2-C3 double bond; has C3-OH	C2	Antioxidant, anti-inflammatory; modulates apoptosis	Quercetin, Kaempferol
Flavanones	Saturated C-ring (no C2-C3 double bond)	C2	General flavonoid activities	Eriodictyol Naringenin
Isoflavones	Similar to flavones	C3	Phytoestrogenic properties	Genistein, Daidzein
Anthocyanins	Positively charged C-ring; glycosylated	C2	Pigmentation; strong antioxidant	Cyanidin, Delphinidin
Flavan-3-ols	Saturated C-ring; has C3-OH	C2	Antioxidant, anticancer	EGCG
Flavanols	Saturated C-ring; no C3-OH/keto group	C2	Reduced form of flavonoids	Catechin
Chalcones	Open-chain (no closed C-ring)	-	Biosynthetic precursor	Isoliquiritigenin
Dihydroflavonols	Saturated C2-C3 bond	C2	Reduced form of flavonols	Taxifolin

Flavonoids are a large family of compounds that can induce programmed cell death through multiple key pathways, such as autophagy, metabolic reprogramming, iron homeostasis, and oxidative stress, thereby exerting their anticancer and antifibrotic activities. Consider lung cancer as a case in point, baicalin induces apoptotic cell death in non-small cell lung cancer (NSCLC) by activating the lysosomal cation channel MCOLN3, which disrupts Ca^2+^ homeostasis, impairs autolysosomal degradation, and ultimately blocks protective autophagy (Dong et al., 2024). Casticin suppresses the proliferation of NSCLC cells by inhibiting glycolytic reprogramming; it downregulates key glycolytic enzymes through the modulation of HIF-1α, thereby targeting the metabolic vulnerability of cancer cells (Wei J. et al., 2025). Quercetagitrin selectively kills NSCLC cells by targeting EIF3D to activate NCOA4-mediated ferritinophagy, which promotes iron-dependent lipid peroxidation and triggers ferroptosis (Tang et al., 2026). The flavonoid GL-V9 induces oxidative stress-mediated apoptosis in small cell lung cancer (SCLC) by binding to and promoting the degradation of STEAP3, which disrupts iron homeostasis and the stability of the mitochondrial protein CISD2 (Zhao et al., 2026).

## 4 Chemical structure and properties of luteolin

Luteolin (3′,4′,5,7-tetrahydroxyflavone) is a flavone consisting of a benzene ring (A) fused to a γ-pyrone ring (C), with a hydroxylated benzene ring (B) attached at the C-ring position 2. The presence of hydroxyl groups at positions 3′, 4′, 5, and seven defines its chemical structure and underlies its key biological activities, including antioxidant effects mediated through free radical scavenging and metal chelation (Zhu et al., 2017). Luteolin demonstrates significant 2,2-diphenyl-1-picrylhydrazyl (DPPH) radical scavenging action, with an IC_50_ of 7.29 µM, comparable to quercetin (4.36 µM) and catechin (5.06 µM) (Zhu et al., 2017). The solubility and bioavailability are affected by glycosylation: luteolin-7-O-β-D-glucopyranoside (galuteolin), extracted from Hibiscus syriacus leaves, exhibits diminished α-glucosidase inhibitory action (IC_50_ 32.12 mg/L) in comparison to aglycone luteolin (IC_50_ 0.6–2.0 µM) (Wei et al., 2015; Zeng et al., 2016). Luteolin’s metal-chelating properties are essential for its antioxidant activity; it effectively binds Fe^2+^ and Cu^2+^ ions, thereby suppressing the Fenton reaction-mediated generation of reactive oxygen species (ROS) (Zhu et al., 2017). Luteolin functions as a non-selective competitive inhibitor of phosphodiesterases (PDEs) one to five, with Kᵢ values between 6.4 and 15.0 µM, hence enhancing its anti-inflammatory and vasodilatory properties (Yu et al., 2010). These properties underlie luteolin’s ability to modulate cellular processes such as EMT, where oxidative stress and inflammation play pivotal roles.

The pharmacokinetic characteristics of luteolin poses obstacles for therapeutic application. *In vitro* investigations indicate that luteolin has favorable intestinal absorption (Caco-2 permeability >10^–6^ cm/s) but possesses low bioavailability owing to significant first-pass metabolism, encompassing glucuronidation and sulfation (Jung et al., 2025). For example, after oral administration of 50 mg/kg luteolin in rats, peak plasma concentration (C_max_) is only 0.3 μg/mL, with a half-life (t_1_/_2_) of 1.2 h (Manzoor et al., 2019). It is worth noting that Glycosylation can influence its bioaccessibility: luteolin-4-O-α-glucoside (L4αG) demonstrates lower water solubility (12.3 μg/mL) and reduced digestive stability (42% retention following simulated gastrointestinal digestion) compared to luteolin-4-O-β-glucoside (L4βG, 25.6 μg/mL solubility, 68% retention). It is worth noting that Glycosylation can influence its bioaccessibility: luteolin-4-O-α-glucoside (L4αG) demonstrates lower water solubility (12.3 μg/mL) and reduced digestive stability (42% retention following simulated gastrointestinal digestion) compared to luteolin-4-O-β-glucoside (L4βG, 25.6 μg/mL solubility, 68% retention) (Jung et al., 2025). However, L4αG shows superior intracellular antioxidant activity, reducing H_2_O_2_-induced ROS by 62% at 50 μM, compared to 48% for L4βG (Jung et al., 2025). Therefore, structural modification of luteolin to enhance its bioavailability is of particular significance.

## 5 Overview of luteolin derivatives

**FIGURE 2:**
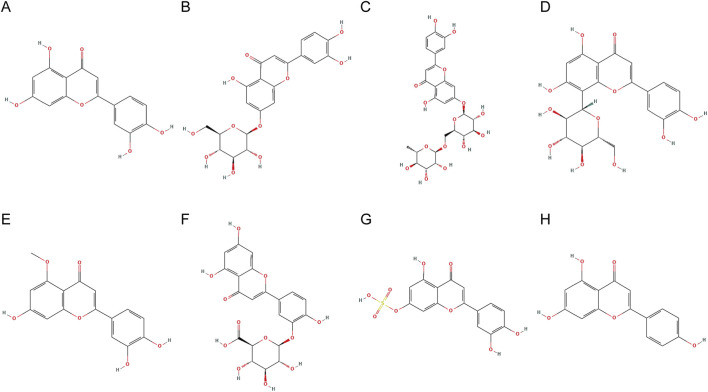
Luteolin and its derivatives. Chemical formula of luteolin **(A)** and its major derivatives **(B-H)**. **(A)** Luteolin **(B)** Luteolin 7-O-glucoside **(C)** Scolymoside **(D)** Orientin **(E)** Luteolin 5-methyl ether **(F)** Luteolin 3′-O-glucuronide **(G)** Luteolin 7-sulfate **(H)** Apigenin.

Due to the poor water solubility and low bioavailability of luteolin themselves, researchers have developed various derivatives through chemical modification or enzymatic reactions to enhance their efficacy. Luteolin derivatives are structurally modified forms of the parent compound, often designed to improve its stability, bioavailability, and specific biological activities. Luteolin derivatives, including glycosylated, methylated, esterified forms, and phospholipid complexes, among others, are rationally designed through structural modifications to overcome the inherent limitations of the parent compound. These modifications significantly enhance water solubility, metabolic stability, bioavailability, and targeted biological activities, thereby improving therapeutic efficacy. The molecular structures of luteolin and its common derivatives are shown in Figure 2 and Table 2.

**TABLE 2:** Luteolin and its derivatives.

Compound	Modification type	Structural significance	Smiles
Luteolin	Parent Aglycone	Features a B-ring catechol group (3′,4′-OH) essential for high redox potential	C1 = CC(=C(C=C1C2 = CC(=O)C3 = C(C=C(C=C3O2)O)O)O)O
Luteolin 7-O-glucoside	O-Glycosylation	Attachment of glucose at the 7-OH position significantly increases hydrophilicity	C1 = CC(=C(C=C1C2 = CC(=O)C3 = C(C=C(C=C3O2)O [C@H]4 [C@@H]([C@H]([C@@H]([C@H](O4)CO)O)O)O)O)O)O
Luteolin 7-rutinoside (Scolymoside)	O-Diglycosylation	Incorporation of a disaccharide (rutinose) at C-7, typical of plant secondary metabolites	C [C@H]1 [C@@H]([C@H]([C@H]([C@@H](O1)OC [C@@H]2 [C@H]([C@@H]([C@H]([C@@H](O2)OC3 = CC(=C4C(=C3)OC(=CC4 = O)C5 = CC(=C(C=C5)O)O)O)O)O)O)O)O)O
Luteolin 8-C-glucoside (Orientin)	C-Glycosylation	A stable C-C bond at C-8 prevents enzymatic cleavage compared to O-glycosides	C1 = CC(=C(C=C1C2 = CC(=O)C3 = C(O2)C (=C(C=C3O)O)[C@H]4 [C@@H]([C@H]([C@@H]([C@H](O4)CO)O)O)O)O)O
Luteolin 5-methyl ether	Methylation	Substitution of the 5-OH with a methoxy group increases lipophilicity	COC1 = CC(=CC2 = C1C(=O)C=C(O2)C3 = CC(=C(C=C3)O)O)O
Luteolin 3′-O-glucuronide	Glucuronidation	A Phase II metabolite formed via conjugation at the 3′position of the B-ring	C1 = CC(=C(C=C1C2 = CC(=O)C3 = C(C=C(C=C3O2)O)O)O [C@H]4 [C@@H]([C@H]([C@@H]([C@H](O4)C (=O)O)O)O)O)O
Luteolin 7-sulfate	Sulfation	Esterification of the 7-OH with sulfate, enhancing systemic clearance	C1 = CC(=C(C=C1C2 = CC(=O)C3 = C(C=C(C=C3O2)OS(=O) (=O)O)O)O)O
Apigenin	Dehydroxylation	Closely related to luteolin but lacks the 3′-OH; this single oxygen atom difference alters biological affinity	C1 = CC(=CC = C1C2 = CC(=O)C3 = C(C=C(C=C3O2)O)O)O

Side chain structure may affect the exposure of these hydroxyl groups and their electron-donating ability, thus influencing the binding mode and affinity of compounds and their derivatives to biomolecules. Luteolin glycosides, generated through microbial biotransformation in hydrophilic organic solvents, are a crucial approach to improve the water solubility and modify the bioactivity of the parent aglycone (Elmowafy et al., 2021). These derivatives, predominantly glycosylated at the C-7, C-3′, or C-4′ hydroxyl positions, including luteolin-7-O-β-glucoside and its di-glucosylated variants, this glycosylation generally enhances the water solubility of a drug, potentially augmenting its absorption and bioavailability *in vivo*, with the 3′,4′-dihydroxy moiety being crucial for antioxidant effectiveness (Xu et al., 2022). Methylated derivatives of luteolin, such as luteolin-5-methyl ether, may alter their metabolic stability and selectivity by introducing methyl groups at the hydroxyl positions. Methylation modification typically reduces the polarity of compounds, which may affect their lipophilicity and transmembrane capacity, thereby impacting their bioavailability and interactions with biological targets (Lin et al., 2008). Sulfated derivatives, including luteolin-3′-O-sulfate, usually increases the polarity of compounds, affecting their metabolic stability and membrane permeability *in vivo* (Kwak et al., 2016; Tan et al., 2022). Other studies have shown that luteolin phospholipid complex (LutPs) significantly enhanced pharmacokinetic performance, achieving a peak plasma concentration of approximately 15,000 nM within 1 h, thereby validating its efficacy in improving luteolin bioavailability through optimized delivery. Synthetic derivatives like benzazepine-conjugated luteolin show potent aldose reductase inhibitory activity (IC_50_ 1.2 µM) and good pharmacokinetic profiles (C_max_ 1.8 μg/mL, t_1_/_2_ 3.5 h) (Sebastian, 2016). Other modifications include acylation, where mono-acylated luteolin derivatives have been synthesized to improve antiproliferative and radical scavenging activities, with implications for enhanced oral bioavailability (Lo et al., 2021). The diverse biological activities of luteolin and its derivatives position them as promising candidates for therapeutic interventions across various diseases, with ongoing research focusing on enhancing their efficacy.

Therefore, the side chain structure of luteolin and its derivatives has a significant impact on solubility, bioavailability, affinity for biological targets, and metabolic stability. Structural modifications of luteolin optimize its pharmacological scaffold, significantly enhancing its efficacy in suppressing the EMT across diverse cancer and fibrosis. The pertinent content will be addressed in the following section.

## 6 Historical perspective on luteolin research

Luteolin’s medicinal use dates back centuries in traditional Chinese medicine (TCM), where plants rich in luteolin, such as Scutellaria baicalensis and Chrysanthemum morifolium, were used to treat inflammation, fever, and liver disorders (Zhang et al., 2016). Modern research on luteolin began in the 1950s with the isolation of its structure and initial studies on its antioxidant properties (Shen et al., 2017). In the 1990s, luteolin’s anticancer effects were first reported, with studies showing inhibition of breast cancer cell proliferation (Imran et al., 2019; Lin et al., 2017). Recent decades have focused on clinical translation and personalized medicine. The current landscape of luteolin research increasingly integrates advanced methodologies, such as bibliometric analysis and network pharmacology, to systematically analyze development trends, knowledge structures, and potential biological mechanisms. This interdisciplinary approach provides insights for its application in nutritional health and disease prevention, solidifying luteolin’s role as a promising multifunctional natural flavonoid (Kumar et al., 2025).

## 7 Luteolin related pathways and biological effects

**FIGURE 3:**
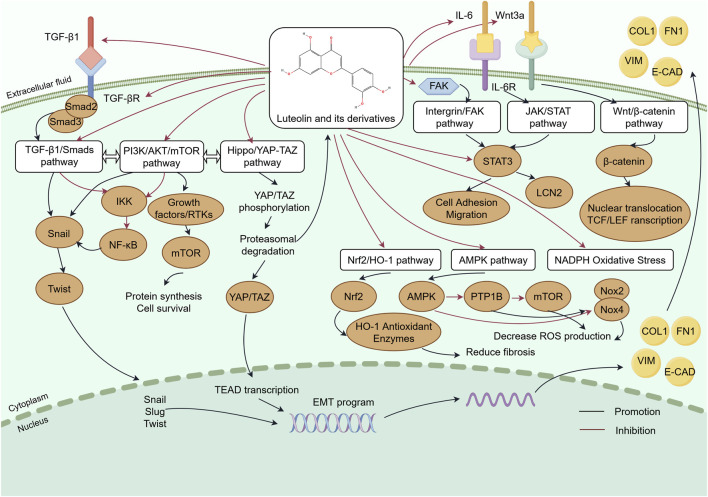
Key signaling pathways and biological functions of luteolin and its derivatives. By Figdraw.

Luteolin exerts synergistic inhibition of EMT via a multi-target, highly interconnected signaling network (Figure 3). Its core mechanism involves the concurrent modulation of three pivotal pathways: TGF-β1/Smad (Li et al., 2023), PI3K/Akt/mTOR (Chai et al., 2024), and Hippo/YAP–TAZ (Zuo et al., 2021). The TGF-β1/Smad pathway represents the most canonical regulator of EMT. By interfering with TGF-β1 signal transduction, luteolin not only suppresses downstream Smad-dependent transcriptional responses but also attenuates key non-Smad signaling branches, including PI3K/Akt and MAPK pathways (Li et al., 2023). Consequently, luteolin inhibits fibroblast activation, blocks initiation of the EMT program, and prevents excessive expression of ECM genes at their transcriptional origin. Luteolin also potently inhibits the PI3K/Akt/mTOR axis, thereby exerting broad downstream effects: it impedes nuclear translocation and transcriptional activity of NF-κB (Gu et al., 2024); downregulates critical EMT-inducing transcription factors—most notably Snail (Zang et al., 2017); and modulates cellular survival and proliferative capacity. This effectively suppresses YAP/TAZ-driven transcriptional programs that are activated by mechanical stress and growth factors, thereby reversing mesenchymal phenotypes and inhibiting cellular invasion (Ghaboura, 2025). Moreover, luteolin suppresses additional pro-EMT signaling modules, including integrin-FAK–mediated mechanotransduction (Ruan JS. et al., 2012; Yang et al., 2024), JAK/STAT3–driven inflammatory signaling (Caporali et al., 2022), and the Wnt/β-catenin pathway (Zhou L. et al., 2025). Collectively, luteolin’s anti-EMT action is orchestrated through this integrated network centered on the aforementioned key pathways.

In addition to directly modulating the aforementioned signaling pathways, luteolin exhibits potent antioxidant activity and thereby indirectly suppresses EMT by restoring cellular redox homeostasis and reprogramming energy metabolism (Zhang et al., 2023; Liu et al., 2021). At the metabolic and stress levels, luteolin could alleviate the pathological process driven by oxidative stress by activating the Nrf2/HO-1 antioxidant pathway, regulating AMPK energy sensing and inhibiting NADPH oxidase-derived reactive oxygen species (Yan et al., 2019). This synergistic inhibition of EMT-associated signaling pathways underlies luteolin’s dual therapeutic effects against tumor metastasis and multi-organ fibrosis, underscoring its promise as a naturally derived, multi-target agent.

It is worth noting that the pharmacological properties of luteolin exhibit a certain degree of tissue specificity. The reasons may be as follows: First, the downstream signaling pathways of luteolin differ. In the lungs, TGF-β1/Smad, FAK/Integrin, and PI3K/AKT/HIF-1α are dominant; in the liver, TGF-β1/Smad and AKT/mTOR are dominant; and in the gastrointestinal tract, Notch/CREB1/Wnt is dominant. Second, the pharmacokinetic changes of luteolin and its derivatives differ in different tissues. For example, luteolin 3′-O-glucuronide and luteolin 7-O-glucoside preferentially accumulate in the liver and intestines, while higher levels of luteolin are retained in the lungs. In addition, the pharmacological properties of luteolin are also related to the microenvironment of different tissues, including oxygen sensitivity (high in the lungs, moderate in the liver, low in GI), ECM stiffness (gradually increased in fibrotic liver and lungs), and immune cell composition (alveolar macrophages, Kupffer cells, and gut-associated lymphoid tissue, respectively). These combined factors explain the differences of luteolin in different tissues.

## 8 Luteolin affects cancer progression through the EMT mechanism

### 8.1 Luteolin’s impact on EMT in breast cancer

Breast cancer constitutes a significant global health burden, with persistently high incidence rates and substantial mortality, particularly evidenced by its status as the most commonly diagnosed cancer among women and a leading cause of cancer-related deaths (Siegel et al., 2025; Xiong et al., 2025). According to the latest research results, the incidence of breast cancer has been slowly increasing since the mid-2000s, and about 316,950 new cases are expected in 2025 (incidence rate: 131.8 per 100,000 women) (Siegel et al., 2025). The pathways and biological effects of luteolin in inhibiting breast cancer are shown in Table 3.

**TABLE 3:** Luteolin inhibits breast cancer pathways and biological effects.

Compound	Target/Pathway	Biological effects	Research type/Experimental validation model	Dosage	Ref
Luteolin	β-catenin signaling pathway	Inhibits migration and invasion of TNBC cells; reverses EMT. Its anti-metastatic effects are mediated by downregulating β-catenin. Expression	*In vitro*:​ Human TNBC cell lines (MDA-MB-231, BT-549); *In vivo*:​ Female nude mouse xenograft metastasis model (subcutaneous injection of MDA-MB-231 cells)	*In vitro*:​ 10 μM,30 μM,100 μM; *In vivo*:​ 100 mg/kg Intraperitoneal injection (i.p.), three times a week for 8 weeks	Lin et al. (2017)
Luteolin	AKT/mTOR signaling pathway; Epigenetic regulation of the MMP9 promoter (H3K27Ac, H3K56Ac)	Inhibits proliferation and metastasis of AR-positive TNBC cells. Reverses EMT. Downregulates MMP9 expression	*In vitro*:​ AR-positive TNBC cell lines	*In vitro*: 10 μM,20 μM,30 μM; IC50 = 13.94 μM	Wu et al. (2021)
Luteolin	YAP/TAZ; Hippo pathway	Suppresses EMT and cell migration in TNBC cells. Inhibits tumor growth *in vivo* by promoting the phosphorylation and proteasome-dependent degradation of YAP/TAZ proteins	*In vitro*:​ Human (MDA-MB-231) and murine (4T1) TNBC cell lines; *In vivo*:​ BALB/c nude mouse xenograft model (subcutaneous injection of 4T1 cells)	*In vitro*:​ 0–80 μM, MDA-MB-231 IC50 = 19.17 μM 4T1 IC50 = 6.98 μM. *In vivo*:​ 40 mg/kg/day, i.p., for 18 consecutive days	Cao et al. (2020)
LU/SL-SDPN (1:1)	STAT3, HIF1-α; Macrophage polarization	Synergistically inhibits STAT3 and HIF1-α; downregulates TGF-β1, CCL2; upregulates TNF-α, iNOS; reprograms TAM from pro-tumor M2 to anti-tumor M1 phenotype	*In vitro*:​ qPCR analysis of M1/M2 markers; In vivo:​ 4T1 tumor-bearing mouse model	*In vitro*:​ 6 μM (LU: 3 μM/SL: 3 μM); *In vivo*: ​80 mg/kg, administered orally by gavage, every other day, for 28 days	Gu et al. (2023)
LUT and CRU	c-Myc, Notch1; IFN signaling, TGF-β1 signaling	Synergistically suppresses TNBC cell proliferation, colony formation, and tumor growth; activates type I IFN signaling; suppresses TGF-β1 signaling; decreases oncoprotein levels of c-Myc and Notch1	*In vitro*:​ MCF7, T47D, BT-549, MDA-MB-231cell line. *In vivo*:​ Xenograft mouse model	*In vitro*:​ LUT EC20: 8.29–44.04 μM, EC50: 34.31–56.94 μM; CRU EC20: 21.66–35.58 μM, EC50: 31.37–46.22 μM. *In vivo*:​ LUT 10 mg/kg/day + CUR 20 mg/kg/day, i.p., every other day for 5 weeks	Wang et al. (2024)

A comprehensive review shows that luteolin, a naturally occurring flavonoid, effectively overcomes breast cancer therapy resistance by potently inhibiting the JAK-STAT signaling pathway and concurrently modulating multifaceted resistance mechanisms, including ATP-binding cassette transporter regulation, EMT suppression, and cancer stem cell targeting, thereby representing a promising candidate for predictive, preventive, and personalized medicine strategies (Kubatka et al., 2025). LIN et al. found that luteolin effectively inhibits triple-negative breast cancer (TNBC) metastasis by reversing EMT, demonstrated through suppressed cell migration, invasion, and reduced lung metastasis *in vivo*. This anti-metastatic action is mechanistically driven by the downregulation of β-catenin, as β-catenin overexpression negates luteolin’s efficacy, establishing a causal pathway through EMT modulation (Lin et al., 2017). In another study of TNBC, Wu et al. found that luteolin suppresses androgen receptor (AR)-positive TNBC metastasis by reversing EMT through inactivation of the AKT/mTOR signaling pathway. This anti-metastatic effect is mediated epigenetically via reduced H3K27Ac and H3K56Ac levels on the MMP9 promoter, thereby downregulating MMP9 expression and inhibiting cell migration and invasion (Wu et al., 2021). In addition, luteolin can inhibits the advancement of TNBC by facilitating the degradation of YAP/TAZ, therefore reversing the EMT, as demonstrated by reduced mesenchymal markers and elevated epithelial markers. The suppression of YAP/TAZ signaling significantly diminishes cell migration *in vitro* and tumor proliferation *in vivo*, establishing luteolin as a potential therapeutic drug aimed at the EMT pathway in TNBC metastasis (Cao et al., 2020). The three aforementioned TNBC investigations indicate that luteolin effectively targets the prevention of breast cancer cell invasion, migration, and metastasis *in vivo*, with a well-defined dose. Research also indicates that luteolin can synergistically enhance anti-breast cancer benefits when combined with other drugs. For instance, the synergistic incorporation of luteolin and silymarin into SDPN nanoparticles (LU/SL-SDPN) can proficiently modulate the conversion of tumor-associated macrophages (TAM) from the pro-tumor M2 phenotype to the anti-tumor M1 phenotype by inhibiting the transcription factors STAT3 and HIF1-α, downregulating M2-related gene expression, and upregulating M1-related gene expression (Gu et al., 2023). Similarly, utilizing xenograft mice models has demonstrated that the concurrent use of luteolin (LUT) and curcumin (CRU) can synergistically impede the proliferation of TNBC. Transcriptomic and proteomic investigations indicated that this synergistic impact arises from the co-activation of the type I interferon signaling route, the suppression of the TGF-β1 signaling pathway, and the downregulation of the oncoproteins c-Myc and Notch1 (Wang et al., 2024). The experiment demonstrated that the amalgamation of LUT and CRU attained superior EC20 (20% effective concentration) and EC50 (50% effective concentration) values. The aforementioned studies indicate that luteolin reverses EMT and suppresses metastasis by targeting pathways including JAK-STAT, β-catenin, and YAP/TAZ; moreover, it works synergistically with other compounds, such as silymarin and curcumin, to augment anti-tumor activity. Consequently, luteolin serves as a promising multi-target option for the treatment of breast cancer.

### 8.2 Luteolin’s impact on EMT in lung cancer

Lung cancer remains the leading cause of cancer-related mortality in the United States, with an estimated 124,730 deaths projected for 2025, accounting for over 20% of all cancer deaths. In the same year, approximately 226,650 new cases are expected to be diagnosed. Although the age-adjusted mortality rate has declined by 61% among men and 38% among women since its peak in the 1990s, lung cancer continues to pose a significant public health challenge, particularly among middle-aged and elderly populations, underscoring its enduring impact on national health outcomes (Siegel et al., 2025). The pathways and biological effects of luteolin in inhibiting lung cancer are shown in Table 4.

**TABLE 4:** Luteolin inhibits lung cancer pathways and biological effects.

Compound	Target/Pathway	Biological effects	Research type/Experimental validation model	Dosage	Ref
Luteolin	WDR72/PI3K-AKT signaling pathway; EMT.	potent anti-tumorigenic effects in NSCLC by directly targeting and downregulating WDR72, which consequently inactivates the PI3K/AKT pathway to suppress tumor growth, metastasis, and EMT.	*In vitro*:​ Human NSCLC cell lines (H1299, A549); *In vivo*:​ Nude mouse subcutaneous xenograft tumor model	*In vitro*:​ 50 μM; *In vivo*:​ 50 mg/kg, i.p., every other day, for 2 weeks	Shi et al. (2025)
Luteolin	AIM2 Inflammasome Pathway	potently suppresses NSCLC progression by inhibiting proliferation, invasion, and EMT while promoting apoptosis. Directly binds and downregulates WDR72, thereby ablating PI3K/AKT signaling to restrict tumor growth *in vivo*	*In vitro*:​ NSCLC cell lines (A549, H460, H226). *In Vivo*:​ A549 and H460 xenograft models in nude mice	*In vitro*:​ 20–80 μM, IC50 = 59.1–81.7μM; *In vivo*:​ 50 mg/kg I.p., every 3 days	Yu et al. (2019)
Luteolin	Integrin β1/Focal Adhesion Kinase (FAK) signaling	Inhibits hypoxia-induced EMT:​ Reverses mesenchymal morphology; restores E-cadherin/ZO-1 (epithelial markers); suppresses N-cadherin, Vimentin, Claudin-1, β-catenin, Snail, TCF/ZEB1 (mesenchymal markers)	*In vitro*​ study. Cell models:​ Human NSCLC cell lines A549 and NCI-H1975. Hypoxia model:​ Chronic exposure to 1% O_2_	*In vitro*: 5, 10, 25, 50 μM	Ruan et al. (2012b)
Luteolin	B7-H3; ADCC	Enhances antitumor immunity of B7-H3-targeted BiKE; boosts ADCC.	*In vitro*:​ ADCC measurement with B7-H3-targeted BiKE. *In vivo*:​ NSCLC xenograft models	*In vitro*:​ 10 μM; *In vivo*:​ 50 mg/kg, i.p. Twice a week	Yang et al. (2026)
Luteolin	PI3K/Akt – NF-κB – Snail signaling. TGF-β1-induced phosphorylation of PI3K/Akt	Inhibits TGF-β1-induced EMT 1. Reverses fibroblast-like morphological changes; 2. Blocks Snail signaling	*In vitro*​ mechanistic study. Primary model: A549 cells. Validation: H460, CL1-0, CL1-5, A549-p53shRNA cells	*In vitro*​: 10 μM,40 μM	Chen et al. (2013)
Luteolin	PRDX2, PD-L1; JAK2/STAT3 pathway	Binds to PRDX2; inhibits JAK2/STAT3 pathway and PD-L1 expression; enhances CD8+ T-cell-mediated cytotoxicity	*In vitro*:​ thermal shift assay, cell proliferation/migration assays; *In vivo*:​ Immunocompetent lung cancer mouse model	*In vitro*:​ 20–50 μM, IC50: 49.53–76.26 μM; 20 mg/kg, i.p., for 18 days	Li et al. (2025b)
Luteolin	AKT1, MAPK3, RAF1/PI3K-AKT, MAPK, NF-κB, VEGF signaling	1. Inhibits cell growth and induces apoptosis 2. Anti-inflammatory and immunomodulatory effects 3. Suppresses EMT mechanisms 4. Inhibits angiogenesis, thereby affecting tumor vascular endothelial cell activity	Network pharmacology analysis, molecular docking validation, molecular dynamics simulation/-	-	Xu et al. (2024)
Luteolin and Apigenin	PD-L1/PD-1 axis; STAT3 Signaling Pathway	Blockade of the PD-1/PD-L1 immune checkpoint has shown anti-tumor activity in KRAS-mutant lung cancer models, and can produce synergistic effects with anti-PD-1 therapy	*In vitro*: KRAS-mutated NSCLC cell line *In vivo*: H358 xenograft tumor (nude mice); Lewis lung cancer model (C57BL/6 J); KRASLA2 genetically engineered mice	*In vitro*: 30μM; *In vivo*: 30 mg/kg, i.p., once a day	Jiang et al. (2021)

Luteolin can influence the progression of lung cancer through multiple mechanisms and pathways. Shi et al. demonstrated that luteolin suppresses EMT in NSCLC through targeting WDR72 and inhibiting the PI3K/AKT signaling pathway. This mechanism was validated both *in vitro* (using molecular docking, functional cellular assays, WB analysis) and *in vivo* (using a subcutaneous xenograft model in nude mice) (Shi et al., 2025). Another study demonstrates that luteolin suppresses the proliferation, invasion, and metastasis of NSCLC in both *in vitro* and *in vivo* models by targeting the downregulation of AIM2 expression, inhibiting its inflammasome activation, and consequently blocking the downstream EMT process (Yu et al., 2019). Similarly, Ruan et al. believe that luteolin markedly inhibits hypoxia-induced EMT in NSCLC cells, as demonstrated by the reinstatement of epithelial markers and the diminution of mesenchymal markers, coupled with the suppression of cell proliferation, motility, and adhesion (Ruan J. et al., 2012). The inhibitory action is partially mediated by the downregulation of the integrin β1/FAK signaling axis, which plays a crucial role in the regulation of EMT. Recent study indicates that B7-H3, an emerging immunological checkpoint molecule overexpressed in NSCLC, is a target for bispecific NK cell binders (BiKE), and its antibody-dependent cytotoxicity (ADCC) is significantly enhanced by luteolin. Luteolin enhances the anti-tumor immunity of B7-H3, synergistically inhibiting the growth of NSCLC and promoting apoptosis both *in vitro* and *in vivo* (Yang et al., 2026). In adenocarcinoma, Other studies have shown that luteolin attenuates TGF-β1-induced EMT in A549 cells, specifically by preventing morphological changes and the downregulation of E-cadherin through the suppression of the PI3K-Akt-IκBa-NF-κB-Snail signaling pathway (Chen et al., 2013). Unfortunately, the above two study did not perform functional validation *in vivo* (Ruan J. et al., 2012; Chen et al., 2013). Luteolin also can bind to peroxiredoxin 2 (PRDX2), inhibits the JAK2/STAT3 pathway to downregulate PD-L1, and promotes CD8^+^ T-cell-mediated cytotoxicity by boosting the production of perforin and granzyme B, therefore reducing the advancement of lung adenocarcinoma in immunocompetent murine models (Li X. et al., 2025). Moreover, research has shown that the detoxifying and gold-clearing formula (JDQJF) impedes the advancement of NSCLC by synergistically targeting critical pathways, including AKT1 and MAPK3, via its various bioactive constituents, such as luteolin, thus modulating EMT-related signaling pathways like PI3K/AKT. Luteolin, as a principal constituent, engages in the regulation of the EMT process, hence indirectly elucidating its functional significance (Xu et al., 2024). It is worth noting that the quality of this study was low because luteolin, as a component in the TCM formula, was not individually targeted, the dose of luteolin was also unknown (Xu et al., 2024). As for luteolin derivatives, Jiang et al. demonstrated that luteolin and its derivative apigenin enhance antitumor immunity in KRAS-mutant lung cancer by suppressing STAT3-mediated PD-L1 expression, thereby synergizing with PD-1 blockade (Jiang et al., 2021). Consequently, luteolin can effectively inhibit the proliferation, invasion, metastasis and epithelial-mesenchymal transition of non-small cell lung cancer *in vitro* and *in vivo* by targeting key molecules such as WDR72, AIM2, B7-H3, PRDX2 and inhibiting PI3K/AKT, JAK2/STAT3 and other signaling pathways. It also enhances anti-tumor immune responses based on immune checkpoints.

### 8.3 Luteolin’s impact on EMT in prostate cancer

Prostate cancer is the most prevalent malignant neoplasm among males in 112 nations, with anticipated cases increasing from 1.4 million in 2020 to 2.9 million by 2040, mostly attributable to demographic aging (James et al., 2024). In the United States, prostate cancer remains the leading incident cancer among men (with an estimated 313,780 new cases in 2025), and its epidemiological burden is further complicated by a concerning shift toward middle-aged populations, underscoring the need for targeted research and intervention strategies (Siegel et al., 2025). The pathways and biological effects of luteolin in inhibiting prostate cancer are shown in Table 5.

**TABLE 5:** Luteolin inhibits prostate cancer pathways and biological effects.

Compound	Target/Pathway	Biological effects	Research type/Experimental validation model	Dosage	Ref
Luteolin	Nrf2-Keap1-Cul3 redox signaling axis	Oxidative stress and apoptosis in mCRPC cells; cytotoxicity and pro-oxidant effects	*In vitro*: PC3, DU145 mCRPC cells and WPMY-1 normal prostate fibroblasts	*In vitro*: 40μM, 80μM, IC50: PC3 = 60μM, DU145 = 65 μM	Eryilmaz et al. (2024)
Luteolin	TFEB; ferritinophagy	Promotes autophagy-dependent ferroptosis in prostate cancer cells	*In vitro*: TFEB knockdown in DU145/PC-3 cells; *In vivo*: nude mouse xenograft model	*In vitro*: 60μM; In vivo: 5 mg/kg/d, i.p. Every 3 days	Fu et al. (2024)
Luteolin	MPO, FOS/TNF signaling pathway, HIF-1 signaling pathway	1. Anti-inflammatory effects 2. Improved metabolic processes 3. Enhanced immune response	Computational Biology and Simulation Research/-	-	Ye et al. (2021)
LUT@ZIF-8	−/−	In prostate cancer (PC3) cells, Luteolin@ZIF-8 exhibits stronger antiproliferative activity and greater anti-migration capacity compared to free luteolin	Construction of nanomedicine delivery systems and *in vitro* efficacy evaluation. Prostate cancer cells: PC3	5 mg luteolin@ZIF-8/25 mL PBS	Ding et al. (2024)

Eryilmaz et al. discovered that luteolin selectively induces oxidative stress and apoptosis in metastatic castration-resistant prostate cancer (mCRPC) cells by inhibiting the Nrf2-Keap1-Cul3 redox signaling axis, as evidenced by dose-dependent cytotoxicity and pro-oxidant assays in PC3 and DU145 cell lines (Eryilmaz et al., 2024). However, its conclusions are not complete due to the lack of *in vivo* experimental validation, which limits the evaluation of its clinical translation potential (Eryilmaz et al., 2024). Furthermore, Luteolin induces ferroptosis in prostate cancer cells by promoting TFEB nuclear translocation and enhancing ferritinophagy, a mechanism validated through inhibitor assays, TFEB knockdown, and *in vivo* tumor models (Fu et al., 2024). Ye et al. propose that luteolin may function as a dual-target therapeutic drug for prostate cancer and COVID-19 b y influencing key genes such as MPO and FOS, which are involved in anti-inflammatory and immune-regulatory pathways. The targeting of these pathways is fundamentally linked to the suppression of the epithelial-mesenchymal transition process, a crucial factor in prostate cancer progression and potentially affecting COVID-19-related tissue pathology (Ye et al., 2021). However, due to the lack of experimental data support, especially the failure to verify its specific regulatory effects on key targets such as MPO and FOS through *in vitro* or *in vivo* models, the strength of evidence for its conclusion is limited. In terms of nanomaterial delivery systems. Studies demonstrate that the encapsulation of LUT into ZIF-8 nanoparticles (LUT@ZIF-8) markedly improves its targeted transport and accumulation in the acidic tumor microenvironment, resulting in more effective prevention of migration in both cervical and prostate cancer cells. The improved anti-migratory effect is directly linked to luteolin’s capacity to reverse the epithelial-mesenchymal transition phenotype, thereby inhibiting the metastatic potential of malignancies (Ding et al., 2024). Consequently, the studies of luteolin in prostate cancer are still relatively limited, the methodological rigor of the existing evidence needs to be improved, and the high concentration used in some studies may cause concerns about PAINS (Bolz et al., 2021). These factors together highlight the need for more in-depth and rigorous research on luteolin.

### 8.4 Luteolin’s impact on EMT in gastrointestinal cancers

Gastrointestinal cancers are a heterogeneous group of malignant diseases that encompass primary tumors of multiple digestive organs, from the esophagus and stomach to the small intestine, colon, and pancreas, posing a significant threat to global public health (Alam et al., 2020; Hanker et al., 2021). The development and progression of gastrointestinal tumors involve complex molecular and pathological mechanisms, including genetic mutations, epigenetic alterations, and interactions with the tumor microenvironment. Despite significant advances in diagnosis and treatment, the global burden of gastrointestinal tumors remains heavy, particularly in the face of challenges such as difficulties in early diagnosis, high tumor heterogeneity, and treatment resistance (Siegel et al., 2025). The pathways and biological effects of luteolin in inhibiting gastrointestinal cancer are shown in Table 6.

**TABLE 6:** Luteolin inhibits gastrointestinal cancer pathways and biological effects.

Compound	Target/Pathway	Biological effects	Research type/Experimental validation model	Dosage	Ref
Luteolin​	Impairs mitochondrial membrane potential (ΔΨm) and inhibits key enzymes of the mitochondrial electron transport chain (METC). The balance of Bcl-2 family proteins (↓Bcl-2/↑Bax)	1. Inhibits proliferation​ of gastric cancer cells. 2. Induces apoptosis​ via the intrinsic pathway. 3. Disrupts mitochondrial function. 4. Impairs membrane-associated ATPase activity​	*In vitro*​:​ Human gastric cancer cell lines HGC-27 and MKN-45; mouse forestomach carcinoma cell line MFC.	*In vitro*​:​ 40 μM, IC50 = 60 μM	Ma et al. (2023)
Luteolin	STAT3 (binds to and inhibits its phosphorylation). STAT3/LCN2 signaling pathway	Alleviates gastric antral atrophy and gastric mucosal damage, inhibits spasmolytic polypeptide-expressing metaplasia (SPEM) to suppress precancerous lesions, and downregulates LCN2 expression	*In vivo*: Tam-induced mice (acute SPEM model); CDCA-induced rats (chronic injury model); *in vitro*: Humanized metaplastic organoids; CDCA-induced cell models	*In vivo*: 20 μM; *in vitro*: 20, 40 mg/kg/d by gavage for 10 days	Hao et al. (2025)
Luteolin	-	In the cohort of patients with stage I-III colorectal cancer, serum luteolin concentrations after diagnosis were not significantly associated with overall mortality, CRC-specific mortality, CRC recurrence, or disease-free survival	Population-based prospective cohort study/Patients diagnosed with stage I-III colorectal cancer in southwestern Germany between 2003 and 2010 (N = 2011)	*In vivo* concentration: 7.39 (5.69, 9.76) ng/μL, Approximate *in vitro* concentration: 25.8 (19.9, 34.1 μM) μM	Jiang et al. (2019)
EA2-PSL-PTX/LUT	Nanocarrier: Specifically targeting CTNNA1 protein on ESCC cells via the EA2 aptamer	It has a significant synergistic anti-tumor effect on ESCC. To alleviate the hepatotoxicity of PTX. It can promote the maturation of dendritic cells and T cell infiltration, reduce MDSCs and Tregs, and reshape the tumor immune microenvironment	Nanomedicine research. *In vitro*: KYSE510, AML12 cell lines. *In vivo*: KYSE150 nude mice xenograft model	*In vitro*: PTX: 7.5 nM + LUT: 15 μM; *In vivo*: PTX 2.5 mg/kg + LUT 10 mg/kg, tail vein injection was given every 7 days for a total of 5 times	Sun et al. (2025a)

In gastric cancer, luteolin inhibits tumor progression primarily by inducing mitochondrial dysfunction and triggering the intrinsic apoptotic pathway—evidenced by loss of mitochondrial membrane potential and impaired electron transport chain activity (Ma et al., 2023). This mitochondria-targeted mechanism concurrently disrupts the bioenergetic and metabolic reprogramming essential for EMT, thereby suppressing the invasive and metastatic capacity of gastric cancer cells (Ma et al., 2023). Hao et al. demonstrate that luteolin directly binds to STAT3 and suppresses its phosphorylation, thereby inhibiting the STAT3/LCN2 signaling axis, a key driver of malignant transformation in gastric precancerous lesions, including the suppression of EMT (Hao et al., 2025). From this, we can conclude that luteolin can not only inhibit the migration and proliferation of gastric cancer cells by inhibiting EMT, but also inhibit the transformation of precancerous lesions. In the field of liver cancer, there is a review elucidates that luteolin exerts its anticancer benefits in liver cancer by altering many essential signaling pathways, including apoptosis and autophagy, which are fundamentally connected to the regulation of EMT, a key driver of metastasis (Rath et al., 2024). However, a prospective cohort study of patients with stage I–III colorectal cancer found no significant association between postdiagnostic serum luteolin levels and clinical prognosis (Jiang et al., 2019). Clinically, the existing evidence does not support blind luteolin supplementation in patients with colorectal cancer who have not received chemotherapy, but further intervention studies are needed to verify (Jiang et al., 2019). Luteolin also modulates the progression of esophageal cancer. As demonstrated by Sun et al., the CTNNA1-targeted, pH-sensitive co-delivery nanocarrier (EA2-PSL-PTX/LUT) significantly enhances the synergistic antitumor efficacy of paclitaxel and luteolin against esophageal squamous cell carcinoma (Sun C. et al., 2025). This is achieved through precise drug release within the acidic tumor microenvironment and potent remodeling of the immunosuppressive tumor milieu, collectively establishing a therapeutic barrier that suppresses EMT-driven invasion and metastasis (Sun C. et al., 2025). The application of nano-delivery system has greatly reduced the dose of luteolin and improved the safety of luteolin. Through the multi-model, multi-method, and multi-control experimental design, the suspicion of PAINS is reduced, and the synergistic anti-tumor effect has high credibility. Although preclinical studies have revealed the potential of luteolin to inhibit tumor progression by targeting pathways associated with EMT and mitochondrial function, and although nanodelivery strategies hold promise for enhancing its targeting and safety, its prognostic value has not been confirmed in clinical cohort studies of colorectal cancer. Therefore, further tumor-specific clinical studies are needed to validate its therapeutic potential.

## 9 Luteolin affects fibrosis progression through the EMT mechanism

### 9.1 Luteolin’s impact on EMT in liver fibrosis

Liver fibrosis, a prevalent development pathway for various chronic liver disorders, presents a substantial public health challenge globally. The epidemiological characteristics and harmful consequences are changing in conjunction with alterations in the etiological mix (Wu et al., 2023; Tao et al., 2020). A systematic review and meta-analysis reveals a substantial global burden of advanced liver fibrosis and cirrhosis, with pooled prevalence rates of 3.3% (95% CI, 2.4%–4.2%) and 1.3% (95% CI, 0.9%–1.7%) in the general population, respectively, which have shown an increasing trend in recent years (Zamani et al., 2025). The advancement of liver fibrosis results in significant liver-related morbidity and mortality. Timely diagnosis and efficient management are essential for enhancing patient outcomes. The pathways and biological effects of luteolin in inhibiting liver fibrosis are shown in Table 7.

**TABLE 7:** Luteolin inhibits liver fibrosis pathways and biological effects.

Compound	Target/Pathway	Biological effects	Research type/Experimental validation model	Dosage	Ref
Luteolin	PTGS2/-	Reduced the expression of α-SMA in activated HSC-T6 cells and inhibited the activation of hepatic stellate cells. Decreased Fibronectin, Col1a1, and Col3a1, and reduced ECM deposition	*In vitro*: TGF-β1-induced HSC-T6 hepatic stellate cell fibrosis model	*In vitro*: 80 μM	Chen et al. (2023)
Luteolin	​CCR1, CD59, NAGA, ITIH3, MKI67, etc.,/affect DNA repair, lysosomal pathway, collagen biosynthesis and other metabolic processes	Reverse the expression changes of a variety of pro-fibrotic/anti-fibrotic proteins induced by TGF-β1 to achieve anti-fibrosis effect	​*In vitro*: TGF-β1-induced fibrosis model of HSC-T6 cells *In vivo*: CCl_4_ induced liver fibrosis rat model	*In vitro*: 2 μM, 10μM, IC50: 15.64 μM; *In vivo*: 10 mg/kg/d by gavage for 3 weeks	Batudeligen et al. (2023)
L7DG	Target: PTP1B /PTP1B-AMPK-mTOR	Inhibit the activation of hepatic stellate cells, downregulate fibrosis markers (Col I, α-SMA, FN1), reduce liver fibrosis and collagen deposition in mice. The indicators of liver injury (ALT, AST) were improved	*In vitro*: TGF-β1-activated LX-2 and primary HSC. *In vivo*: CCl_4_ -induced and dietary + CCl_4_ -induced liver fibrosis/NASH mouse models	*In vitro*: 50 μM, IC50 = 2.10 ± 0.26μM; *In vivo*: A single dose of 40–150 mg/kg/d was administered by gavage	Tang et al. (2025)
LUT-Ex	-/TGF-β1/Smad, AKT/mTOR, and STAT3 pathways	Compared with free LUT, the LUT-Ex exhibited enhanced cellular uptake and superior anti-fibrotic activity *in vivo*	*In vitro*: Cellular uptake assay in HEP-G2 cells *In vivo*: Liver fibrosis rat model (male Sprague-Dawley rats)	In vitro: 7 mg/mL; In vivo: 1.4 mg/kg, single i.p.; The dose of exosome carrier was 450 μg/kg	ASHOUR et al. (2022)
LUT@LIP-BSA and CMB	aHSCs targeted via BSA binding to SPARC receptor	Regulate the Keap1/Nrf2 pathway, the cGAS-STING pathway, p53/Bcl-2 pathway, and inhibits TGF-β1 signaling to reduce α-SMA and COLI expression	In vitro: HSC-T6 and AML-12 cell models In vivo: CCl4-induced liver fibrosis model in ICR mice	*In vitro*: 50 μM; *In vivo*: 2 mg/kg twice a week by tail vein injection	Sun et al. (2025b)

Luteolin exerts anti-hepatic fibrosis effects by targeting key pathways such as PTGS2, MAPK, and AKT1 (Chen et al., 2023). It significantly reduces expression of fibrotic markers (COL1a1, α-SMA, PC-III, etc.) in hepatic stellate cells (HSC) cells, thereby inhibiting ECM deposition and hepatic stellate cell activation (Chen et al., 2023). This article is a screening of active ingredients in Lamiophoromis Herba (LH). It only reports the dosage of luteolin in vitro and does not involve *in vivo* or clinical studies. Furthermore, the dosage of luteolin is relatively large, and PAINS interference cannot be ruled out. Therefore, the quality grade is low. Further research indicates luteolin target action entails the upregulation of antifibrotic proteins (CCR1, CD59) and simultaneous downregulation of profibrotic mediators (P4HA3, FBLN2, MKI67), which collectively inhibit ECM biosynthesis, collagen organization, and abnormal cell migration, thereby mitigating the fibrogenic phenotype, however, its downstream mechanisms require further investigation (Batudeligen et al., 2023). This study elucidates the molecular network underlying luteolin’s anti-fibrotic effects, identifying specific targets and mechanisms, thereby providing a basis for the development of anti-fibrotic therapies. As for derivatives of luteolin, Tang et al. discovered that luteolin derivative Luteolin-7-diglucuronide (L7DG) mitigates liver fibrosis chiefly by inhibiting protein tyrosine phosphatase 1 B (PTP1B), resulting in the activation of AMPK. This cascade simultaneously inhibits the classical activation of HSC and suppresses the TGF-β1-driven EMT program, thereby reducing the expression of fibrogenic markers and ECM deposition, which demonstrates that luteolin derivatives also exhibit anti-fibrotic effects (Tang et al., 2025). In this study, the dose of 150 mg/kg/d in mice was approximately equal to 24.3 mg/kg/day in humans, which was within the safe dose range for oral administration and had the potential for clinical transformation. Ashour et al. first developed luteolin loaded mesenchymal stem cell-derived exosomes (LUT-Ex), which showed superior anti-liver fibrosis activity than free luteolin both *in vitro* and *in vivo*, however, no clear target was given in this study (Ashour et al., 2022). An efficient delivery system is equally important. Employing an activated hepatic stellate cells (aHSCs)-targeted dual-delivery system which composed of luteolin-loaded liposomes (LUT@LIP-BSA) and Ce/Mn bimetallic nanozymes (CMB), luteolin can reverse liver fibrosis by inducing senescence and apoptosis while counteracting oxidative stress in activated hepatic stellate cells (Sun Z. et al., 2025). This experiment showed that LUT@LIP-BSA was highly enriched in the liver, and colocalization with aHSCs markers was detected. Consequently, while luteolin and its derivatives have shown anti-fibrotic potential through targeting key pathways and advanced delivery systems, further *in vitro* data are needed to assess their clinical translational potential.

### 9.2 Luteolin’s impact on EMT in renal fibrosis

Renal fibrosis represents the ultimate common pathologic process in the progression of chronic kidney disease (CKD) to end-stage renal disease (ESRD). It is characterized by abnormal deposition of ECM within the renal parenchyma, leading to structural disruption and functional loss of the kidney. The rising global prevalence of chronic kidney disease poses a significant public health challenge. The epidemiology of renal fibrosis highly overlaps with CKD, affecting approximately 9.1% of the population (equivalent to 697.5 million cases). It ranks as the third leading cause of premature death, posing a serious threat to human health (Wei et al., 2024). The pathways and biological effects of luteolin in inhibiting renal fibrosis are shown in Table 8.

**TABLE 8:** Luteolin inhibits renal fibrosis pathways and biological effects.

Compound	Target/Pathway	Biological effects	Research type/Experimental validation model	Dosage	Ref
Luteolin	Nr4a1-Slc7a11-GPX4 pathway/Ferroptosis	​To improve the renal function and histopathological damage induced by calcium oxalate (CaOx) crystals, inhibit ferroptosis, and reduce renal injury and fibrosis	*In vitro*: CaOx crystal-treated HK-2 human renal tubular epithelial cells. *In vivo*: Glyoxylate-induced CaOx kidney stone model in mice	*In vitro*: 100μM; 100, 200 mg/kg/day, i.p., for 12 consecutive days	Ye et al. (2025)
Luteolin	SIRT1/FOXO3 pathway.​ Binds to and activates SIRT1	1. Increases hemoglobin, hematocrit, and erythropoietin (EPO) levels 2. Alleviates renal fibrosis: Reduces blood urea nitrogen, creatinine, and fibrosis markers 3. Upregulates HIF2A and SIRT1/FOXO3: Modulates the hypoxia response and cellular stress resistance pathways	​*In vitro*: hypoxia-reoxygenation-induced renal interstitial fibroblast fibrosis model in NRK49 F rats. *In vivo*: aristolochic acid-induced renal interstitial fibrosis and renal anemia mouse model	*In vitro*: 10, 20μM; In vivo: 50, 100 mg/kg/d by gavage for 4 weeks	Li et al. (2022)
Luteolin	Nrf2/HO-1 signaling pathway	Reduce acute kidney injury (AKI) and inhibit its fibrosis progression to CKD. It can inhibit oxidative damage and inflammatory apoptosis, downregulate inflammatory markers and regulate the expression of apoptosis-related proteins. Inhibition of tissue fibrosis induced by I/R injury	*In vivo*: Rat model of AKI-to-CKD transition induced by unilateral ischemia/reperfusion injury (UIRI)	*In vivo*: 50 mg/kg/d by gavage for 30 days	Wei et al. (2025b)

Ye et al. demonstrate that luteolin attenuates CaOx crystal-induced renal fibrosis primarily by inhibiting Nr4a1-dependent ferroptosis (Ye et al., 2025). It abrogates the activation of the downstream EMT program in tubular epithelial cells, thereby reducing ECM deposition and preserving renal architecture. Another study shows that via stimulating the SIRT1/FOXO3 axis, luteolin reduces renal anemia brought on by interstitial fibrosis. Concurrently, SIRT1/FOXO3 activation increases the generation of erythropoietin and decreases the deposition of extracellular matrix, both of which aid in the recovery of hematological parameters and renal function (Li et al., 2022). In renal fibrosis induced by ischemia–reperfusion (I/R) injury, luteolin ameliorates EMT through activation of the Nrf2/HO-1 antioxidant signaling pathway, thereby preserving tubular epithelial integrity and suppressing pathological extracellular matrix accumulation (Wei JP. et al., 2025). Although these preliminary studies have revealed the potential of luteolin to alleviate renal fibrosis through multiple signaling pathways, the experimental studies are limited in number and vary in quality, and their clinical relevance remains unclear; further rigorous research is needed to validate these findings.

### 9.3 Luteolin’s impact on EMT in cardiac fibrosis

Cardiac fibrosis is a pathological process characterized by excessive accumulation of ECM components, particularly collagens, within the myocardial interstitium and perivascular spaces (Li et al., 2024). Cardiac fibrosis represents a common endpoint of diverse cardiac insults, including myocardial infarction, pressure overload, diabetes mellitus, and aging, and constitutes a major contributor to heart failure progression and arrhythmogenesis (Han et al., 2023). The underlying mechanism involves activation of resident cardiac fibroblasts into myofibroblasts. Globally, the epidemiology of myocardial fibrosis displays substantial heterogeneity and a progressive annual increase. Depending on the underlying cardiac disease subtype and the imaging modality employed, the prevalence of myocardial scarring in asymptomatic or mildly symptomatic patients with hypertrophic cardiomyopathy has also shown a marked upward trend (Zhang et al., 2020). The pathways and biological effects of luteolin in inhibiting cardiac fibrosis are shown in Table 9.

**TABLE 9:** Luteolin inhibits cardiac fibrosis pathways and biological effects.

Compound	Target/Pathway	Biological effects	Research type/Experimental validation model	Dosage	Ref
Luteolin	NF-κB and Nrf2 signaling pathways	Reduce myocardial fibrosis and apoptosis in diabetic cardiomyopathy by inhibiting NF-κB-mediated inflammatory pathway and activating NrF2-mediated antioxidant response	*In vitro*:​ High glucose (HG)-induced injury model in H9C2 rat cardiomyocyte cell line. *In vivo*:​ STZ-induced DCM C57BL/6 model	*In vitro*:​ 5 μM,10 μM; *In vivo*:​ 20 mg/kg/day, by gavage, once daily for 15 weeks	Li et al. (2019)
Luteolin	TGF-β1/CTGF signaling pathway; NADPH oxidase (Nox2, Nox4) -mediated oxidative stress; JNK MAPK signaling pathway	1. Reduce angiotensin II (Ang II) -induced cardiac fibrosis and hypertrophy 2. Reduce oxidative stress level in cardiac tissue 3. Inhibit the expression of TGF-β1 and the phosphorylation of JNK in cardiac fibroblasts induced by H_2_O_2_ *in vitro*	*In vitro*: cultured rat cardiac fibroblasts (H_2_O_2_ stimulated); *In vivo*: Ang II-induced hypertension in Sprague-Dawley rats	10 μM,20 μM; 0.035% feed, mixed into the basal diet, is given to rats free access	Nakayama et al. (2015)
L7DG	NADPH oxidase subunits (Cyba, Cybb, Ncf1, Ncf4, Rac2); TGF-β1 signaling pathway	L7DG effectively antagonizes ISO-induced myocardial injury and fibrosis *in vivo* by inhibiting NADPH oxidase-mediated oxidative stress and regulating TGF-β1 signaling pathway and related miRNA expression	*in vivo*:​ ISO-induced myocardial injury and fibrosis model in C57BL/6 J mice	*in vivo*:​ 5, 10, 20, and 40 mg/kg/day, i.p., for 5 days	Ning et al. (2017)

Luteolin also exerts inhibitory effects on cardiac fibrosis. Li et al. used streptozotocin (STZ) to create a model of diabetic cardiomyopathy (DCM) and found that luteolin ameliorates cardiac fibrosis in DCM by suppressing NF-κB-mediated inflammatory responses and activating Nrf2-driven antioxidant pathways, thereby reducing ECM deposition and cellular hypertrophy, which potentially involve the inhibition of EMT (Li et al., 2019). Research also indicates luteolin exerts its cardioprotective effects primarily by attenuating oxidative stress, thereby suppressing the expression of key NADPH oxidase subunits (NOX2/NOX4) and subsequent ROS production. This reduction in oxidant signaling inhibits the TGF-β1/CTGF fibrotic pathway and JNK phosphorylation, ultimately decreasing the expression of EMT markers (COL1, COL3), which mitigates both cardiac fibrosis and hypertrophy (Nakayama et al., 2015). It is worth noting that this study used a luteolin-mixed feed feeding method instead of the traditional gavage method. This method is simple to operate and reduces animal stress, but it has limitations such as uneven individual intake and insufficient dosage accuracy. Moreover, L7DG, a derivative of luteolin, blocks the initiation of myocardial fibrosis by reducing oxidative stress through inhibiting the expression of NADPH oxidase subunit genes. Concurrently, L7DG downregulates collagen and ECM gene expression while reversing TGF-β1 signal related microRNA abnormalities, thereby comprehensively suppressing the fibrotic process (Ning et al., 2017). Collectively, these results indicate that luteolin and its derivative L7DG mitigate cardiac fibrosis via several mechanisms; nonetheless, there is a paucity of relevant literature, necessitating more investigation.

### 9.4 Luteolin’s impact on EMT in pulmonary fibrosis

Pulmonary fibrosis (PF) is a chronic, progressive, and frequently lethal interstitial lung disease marked by the irreversible buildup of scar tissue in the lungs, resulting in impaired gas exchange and respiratory failure (Mohanan et al., 2024). Idiopathic pulmonary fibrosis (IPF) is the most prevalent and severe variant of PF, characterized by a distinct pattern of typical interstitial pneumonia (UIP) observed using high-resolution computed tomography (HRCT) and, in certain instances, lung biopsies (Luppi et al., 2021; Podolanczuk and Raghu, 2024; Raghu et al., 2022). Incidence rates for IPF range from three to nine per 100,000 person-years, while prevalence estimates are typically between 13 and 20 per 100,000 in regions such as North America and Europe (Luppi et al., 2021). The pathways and biological effects of luteolin in inhibiting PF are shown in Table 10.

**TABLE 10:** Luteolin inhibits pulmonary fibrosis pathways and biological effects.

Compound	Target/Pathway	Biological effects	Research type/Experimental validation model	Dosage	Ref
Luteolin	AMPK/PPAR-γ signaling pathway	Inhibits fibroblast proliferation and myofibroblast differentiation	*in vitro*: TGF-β1-treated human lung fibroblast MRC-5 cell line	*in vitro*: 5μM, 10μM, 20 μM	Yan et al. (2023)
LG	TGF-β1 protein TGF-β1/Smad (p-Smad2/3, Smad2/3); TGF-β1/non-Smad (ERK1/2, p38) signaling	1. Inhibition of fibroblast activation 2. Inhibition of FMT 3. Enhanced cell adhesion 4. Reduce ECM degradation and deposition	*In vitro*: Mouse fibroblasts (L929) stimulated with TGF-β1	*in vitro*: 12.5 μg/mL, 25 μg/mL, 50 μg/mL; 27μM, 54μM, 108 μM	Peng et al. (2025)
LUT@CDMOF	IL-6, IL-1β, TNF-α pathways	Reduces pro-inflammatory cytokine levels and inhibits fibrosis progression in the lung	*In vitro*: Alveolar macrophage (MHS) cell model. *In vivo*: BLM induced PF rat model	5 mg/kg, Microspray, once daily	Ren et al. (2023)
LUT@HAase	IL-6, IL-1β, TNF-α pathways and TGF-β1 induced α-SMA and fibronectin	Inhibits fibrosis, reduces inflammation and ROS, improves lung function and survival	*In vitro*: MRC5 cell model. *In vivo*: BLM-induced mouse model	Nebulized inhalation, once every 3 days, for a total of five doses	Pan et al. (2025)

Yan et al. used an orthogonal experimental design to quantitatively verify the effect of luteolin in the Renshen Pingfei Formula (RSPF) active ingredients on inhibiting myofibroblast differentiation by activating the AMPK/PPAR-γ pathway in a TGF-β1-induced MRC-5 human embryonic lung fibroblast (Yan et al., 2023). Although this experiment was based on compound active ingredients, the analysis of luteolin monomers has certain reference value. Furthermore, the anti-fibrotic action of luteolin derivatives (Luteolin 7-Glucuronide, LG) is mediated by a dual mechanism: direct inhibition of the TGF-β1 signaling pathway through targeted binding, and suppression of oxidative stress by downregulating NADPH oxidase. This converges to inhibit fibroblast activation and transition (EMT/FMT), thereby reducing the pathological deposition of ECM proteins (Peng et al., 2025), however, *in vivo* efficacy validation for monomers is lacking. An advanced delivery mechanism can also improve medicinal effectiveness. Ren et al. demonstrates that a cyclodextrin metal-organic framework (CD-MOF)-based dry powder inhaler delivery system for luteolin (LUT@CDMOF) significantly enhances its pulmonary deposition and bioavailability, effectively ameliorating bleomycin (BLM)-induced fibrosing interstitial lung disease in rats (Ren et al., 2023). In another investigation, the inhalation of hyaluronidase nanoparticles encapsulating luteolin (LUT@HAase) also successfully traversed the drug delivery barrier in PF tissue, resulting in profound penetration (Pan et al., 2025). This study innovatively employs a nebulized inhalation route of administration, providing a new strategy for PF; however, the lack of clearly defined LUT@HAase concentrations limits reproducibility and clinical translation. Collectively, novel delivery systems (such as CD-MOF and HAase nanoparticles) have enhanced the lung targeting of luteolin, providing a promising approach for PF.

## 10 Nanoformulation of Luteolin for fibrosis and cancer

Nanotechnology and Nanocarriers are increasingly acknowledged as a potential approach for the precise transport of pharmaceuticals to targeted sites within organs, tissues, or cells, assuring the safe administration of therapeutic agents (Watkins et al., 2015; Zhou et al., 2023; Rana et al., 2025). The integration of natural goods with nanotechnology presents substantial promise, since nanoparticles facilitate the encapsulation of one or more natural chemicals, commonly used nanomaterials include nanoparticles, liposomes, and nanoemulsions (Rana et al., 2025; Akter et al., 2025). The application of nanoscale drug delivery systems to luteolin can substantially improve its bioavailability, enable targeted delivery, and facilitate controlled release (Singh and Shukla, 2025). This article mentions several nanodelivery systems, such as LUT@ZIF-8, LUT@LIP-BSA, and LUT@CDMOF. These delivery systems overcome the pharmaceutical drawbacks of luteolin, such as poor water solubility, low bioavailability, and rapid metabolism, significantly improving its targeting, therapeutic efficacy, and clinical application potential (Ding et al., 2024; Pan et al., 2025). While nanodelivery systems offer many advantages, their biosafety, *in vivo* stability, long-term toxicity, and feasibility for large-scale production require further investigation. Furthermore, precisely controlling the release behavior of nanoparticles is also an important direction for future research (Pan et al., 2025). With the development of nanotechnology, it is hoped that more effective and safer luteolin nanomedicines can be developed, ultimately enabling their widespread clinical application.

## 11 Addressing PAINS considerations for luteolin and its derivatives

PAINS are defined as chemical entities that produce false-positive results in biological assays via non-specific, promiscuous mechanisms, including compound aggregation, non-selective redox cycling, fluorescence quenching, non-specific covalent modification, or metal chelation unrelated to target binding (Bolz et al., 2021). Luteolin, a flavone with a 3′,4′-dihydroxy catechol moiety on its B-ring, may trigger *in silico* PAINS alerts due to this structural feature. However, the extensive literature synthesized in this review provides evidence that the key targets of luteolin and its derivatives differ from the nonspecific confounding defined by PAINS.

First, all the studies we included showed a dose-response relationship: regardless of *in vitro* (5–100 μM) or *in vivo* (5–200 mg/kg), the anti-EMT activity of luteolin showed a predictable trend with increasing exposure, unlike the non-specific false positive signal across the entire concentration range of PAINS. Second, the target effect of luteolin has been clearly validated: multiple studies have confirmed through surface plasmon resonance (SPR) (Cao et al., 2025; Deng et al., 2026; Gu and Pang, 2025) and cellular thermal shift analysis (CETSA) (Cao et al., 2025; Cheng et al., 2025; Liu et al., 2025) that it can directly and specifically bind to core targets such as TGF-β1, ruling out the possibility of non-specific aggregation or fluorescence interference. Third, the *in vivo* efficacy of luteolin has been reproducibly validated in multi-organ fibrosis and cancer models involving the liver, kidneys, lungs, and heart. Furthermore, studies on different routes of administration (gavage, intraperitoneal injection, and nebulized inhalation) and formulation optimization have confirmed a clear correlation between *in vivo* exposure and efficacy. Simultaneously, luteolin derivatives modified with different chemical structures exhibit predictable differences in solubility, bioavailability, and target affinity (structure-activity relationship, SAR), which is unattainable by PAINS compounds with non-specific effects. In fact, early PAINS screening algorithms had a significant inherent misclassification rate for natural products, with many flavonoids being misclassified as interfering substances due to their phenolic hydroxyl structure. However, their true pharmacological activities have been confirmed by numerous studies, and these well-mechanistically validated natural products cannot be classified as PAINS solely based on algorithmic predictions.

## 12 Immune reprogramming and future prospects

In addition to influencing the EMT mechanism in parenchymal cells, luteolin can also act on the shared immune microenvironment of cancer and fibrosis, thereby eliminating abnormal cells through immune surveillance. For example, luteolin repolarizes M2 macrophages (including TAM in cancer and pro-fibrotic macrophages in fibrosis) into M1 macrophages, thereby clearing malignant and overactivated cells. In fibrosis, myofibroblasts can activate interferon signaling to drive CD8^+^ cytotoxic T cell infiltration, while reversing T cell exhaustion through STAT3-dependent PD-L1 downregulation and disrupting the systemic immunosuppressive network by reducing the accumulation of regulatory T cells and myeloid-derived suppressor cells and attenuating TGF-β1-mediated immunosuppression. These synergistic immune regulatory axes restore host immune surveillance, explaining the broad efficacy of luteolin in various cancer and fibrosis.

Future work should prioritize clinical translation of luteolin-based delivery systems, along with large-scale clinical trials to validate their efficacy in fibrosis-cancer comorbidities. Rigorous pharmacodynamic validation would address concerns about target specificity.

## 13 Conclusion

In summary, luteolin and its derivatives are multi-target modulators of fibrosis-cancer, exerting their effects through an anti-EMT mechanism. Luteolin simultaneously targets key signaling pathways such as TGF-β1/Smad, PI3K/Akt/mTOR, and Hippo/YAP-TAZ, while also targeting Nrf2/HO-1 to restore redox homeostasis. Luteolin reverses the mesenchymal phenotype to inhibit metastatic spread in breast, lung, and gastrointestinal cancers, and can alleviate pathological ECM deposition, thereby improving fibrosis in the liver, kidneys, heart, and lungs, exhibiting consistent, dose-dependent efficacy across different disease contexts. Crucially, specific structural modifications and nanodelivery systems effectively overcome the limitations of poor bioavailability and rapid metabolism of the parent compound. Furthermore, rigorous validation of target binding, reproducible *in vivo* efficacy, and a clear SAR exclude PAINS, confirming the specific pharmacological activity of luteolin. These findings contribute to the clinical application of luteolin for fibrosis and cancer, and future efforts focus on advancing the optimized formulation into rigorous clinical trials to unlock its full therapeutic potential.

## Glossary

EMT: Epithelial-Mesenchymal Transition
ECM: Extracellular Matrix
TGF-β1: Transforming Growth Factor Beta one
α-SMA: Alpha-Smooth Muscle Actin
COL1A1: Collagen Type I Alpha 1 Chain
PI3K: Phosphatidylinositol 3-Kinase
Akt: Protein Kinase B
mTOR: Mammalian Target of Rapamycin
Hippo: Hippo Signaling Pathway
YAP: Yes-associated Protein
TAZ: Transcriptional Co-activator with PDZ-binding Motif
STAT3: Signal Transducer and Activator of Transcription three
PD-L1: Programmed Death-Ligand one
MMP9: Matrix Metallopeptidase nine
PTP1B: Protein Tyrosine Phosphatase 1B
AMPK: AMP-activated Protein Kinase
Nrf2: Nuclear factor erythroid 2-related factor 2
HO-1: Heme Oxygenase-1
TME: Tumor Microenvironment
CAFs: Cancer-Associated Fibroblasts
NSCLC: Non-Small Cell Lung Cancer
SCLC: Small Cell Lung Cancer
TNBC: Triple-Negative Breast Cancer
AR: Androgen Receptor
TAM: Tumor-Associated Macrophage
IPF: Idiopathic Pulmonary Fibrosis
CKD: Chronic Kidney Disease
ESRD: End-stage Renal Disease
HSC: Hepatic Stellate Cell
aHSCs: Activated Hepatic Stellate Cells
I/R: Ischemia/Reperfusion
BLM: Bleomycin
PAINS: Pan-Assay Interference Compounds
i.p: Intraperitoneal Injection
S.c.: Subcutaneous Injection
Cmax: Peak Plasma Concentration
T1/2: Half-life
IC50: Half Maximal Inhibitory Concentration.
EC50: Half Maximal Effective Concentration
